# The relationship between body composition and left ventricular performance in women with breast, lymphoma, or sarcoma cancer

**DOI:** 10.1186/s40959-024-00233-1

**Published:** 2024-06-06

**Authors:** Leila Mabudian, Kerry Reding, Ralph B. D’Agostino, Emily M. Heiston, Moriah P. Bellissimo, Kristine Olson, William O. Ntim, Heidi D. Klepin, Emily V. Dressler, Tonya Moore, Jennifer H. Jordan, Nathaniel S. O’Connell, Amy Ladd, Kathryn E. Weaver, Bonnie Ky, Lynne I. Wagner, Mary Helen Hackney, Glenn J. Lesser, W Gregory Hundley, The UPBEAT Study Team

**Affiliations:** 1 Division of Cardiology, VCU Pauley Heart Center, Virginia Commonwealth University (VCU), PO Box 980335, Richmond, VA USA; 2 University of Washington, Behavioral Nursing and Health Systems, Seattle, WA USA; 3 Department of Biostatistics and Data Science, Wake Forest University School of Medicine, Winston-Salem, North Carolina, USA; 4 UNC School of Medicine, Novant Health Campus, Novant Health Heart & Vascular Institute, Charlotte, NC USA; 5 Section On Hematology and Oncology, Department of Internal Medicine at Wake Forest School of Medicine, Winston-Salem, North Carolina USA; 6 Section On Cardiovascular Medicine, Department of Internal Medicine at Wake Forest School of Medicine, Winston-Salem, North Carolina USA; 7 Department of Biomedical Engineering, Virginia Commonwealth University (VCU), Richmond, VA USA; 8 Department of Social Sciences and Health Policy, Wake Forest School of Medicine, Winston-Salem, North Carolina USA; 9 Department of Medicine, Perelman School of Medicine at the University of Pennsylvania, Philadelphia, PA USA; 10 Department of Hematology, Oncology, and Palliative Care, Virginia Commonwealth University, Massey Cancer Center, Richmond, VA USA; 11 Department of Health Policy and Management, Gillings School of Global Public Health, University of North Carolina, Chapel Hill, North Carolina USA

**Keywords:** Body mass index, Cardiovascular risk, Heart failure, Adipose tissue, Abdominal obesity

## Abstract

**Background:**

To understand how body composition in those with elevated body mass index impacts left ventricular function decline during cancer treatment, we determined the association between baseline body mass index (BMI), intra-abdominal adipose tissue (IAT) and subcutaneous adipose tissue (SAT) with baseline to 3-month left ventricular ejection fraction (LVEF) change among women receiving potentially cardiotoxic chemotherapy for breast cancer, lymphoma, or sarcoma.

**Methods:**

Women underwent potentially cardiotoxic chemotherapy, such as doxorubicin, cyclophosphamide, paclitaxel, and trastuzumab, for treatment of breast cancer, lymphoma, or sarcoma. We obtained magnetic resonance images (MRIs) of body composition and cardiac function prior to treatment, and then a repeat MRI for cardiac function assessment at three months into treatment. Analyses and assessment of abdominal adipose tissue volumes and LVEF outcomes were conducted by independent reviewers blinded to all patient identifiers. A general linear model was created to examine associations between adipose tissue depots, BMI, and 3-month LVEF change.

**Results:**

Women (*n* = 210) aged 56 ± 11 years with breast cancer, lymphoma, and sarcoma were enrolled (*n* = 195, 14, 1 respectively). Baseline BMI, IAT, and SAT fat were independently associated with 3-month LVEF declines (*p* = 0.001 to 0.025 for all). After adjusting for baseline cardiovascular disease risk factors, BMI, IAT, and SAT, BMI remained the only variable associated with 3-month LVEF decline (*p* = 0.047).

**Conclusions:**

These results suggest that factors other than abdominal adipose tissue or traditional cardiovascular risk factors may contribute to 3-month declines in LVEF among women with elevated BMI receiving potentially cardiotoxic chemotherapy. Further investigation should be conducted on psychosocial stress, physical activity, sleep, or diet.

**Trial registration:**

DETECTIV_NCT01719562, WF99112, & WF97415—NCT02791581.

## Background

The development of heart failure accompanied by declines in left ventricular (LV) ejection fraction is accelerated in patients receiving potentially cardiotoxic chemotherapy for cancer treatment [1]. Obesity, defined as a body mass index (BMI) ≥ 30 kg/m [2], is a risk factor for heart failure and LV dysfunction upon receipt of potentially cardiotoxic chemotherapy [2, 3]. However, not all research has observed this association between BMI and changes in left ventricular ejection fraction (LVEF) upon receipt of chemotherapy. Previously, we noted that intraperitoneal fat but not BMI was associated with 24-month LVEF declines in men and women receiving potentially cardiotoxic chemotherapy (*r* = -0.33, *p*= 0.02) [4].

Accordingly, the goal of this study was to examine if intra-abdominal adipose tissue (IAT) was associated with BMI and LVEF decline in a cohort of women receiving potentially cardiotoxic chemotherapy for breast cancer, lymphoma, or soft tissue sarcoma.

## Methods

### Study design

This prospective cohort study (NIH R01CA167821 & R01CA199167) was conducted in collaboration with the Wake Forest National Cancer Institute Community Oncology Research Program Research Base and ECOG-ACRIN Cancer Research Group under a National Cancer Institute CIRB-approved protocol and a Wake Forest University School of Medicine (WFU SOM UG1CA189824, ECOG-ACRIN UG1CA189828) IRB approved protocol. All participants provided written informed consent.

At baseline, a cardiac and abdominal magnetic resonance imaging (MRI) were obtained. Three months after the baseline visit, a second cardiac MRI was obtained. The 3-month interval allows for a reasonable timeframe to observe the effects of treatment on LVEF without delaying clinical decision-making. Using the 3-month cardiac MRI, LVEF change from baseline to 3 months was calculated, while the abdominal MRI measured baseline IAT (combination of retroperitoneal [RP] and intraperitoneal [IP] adipose tissue depots) and subcutaneous adipose tissue (SAT). All images were analyzed by readers who were blinded to all patient identifiers.

### Study population

Female cancer patients scheduled to receive potentially cardiotoxic chemotherapy from January 2013 to July 2020, were recruited from participating sites. Females were included if they were a) scheduled to receive potentially cardiotoxic chemotherapy for breast (stages I-III), lymphoma (stages I-IV), or soft tissue sarcoma cancer, b) aged > 21 years, c) exhibited a pre-cancer treatment LVEF > 50%, d) had a life expectancy of > 2 years, and e) underwent a pre-cancer treatment MRI of the abdomen along with a cardiac MRI before and 3 months after initiating treatment. Doxorubicin, cyclophosphamide, paclitaxel, and trastuzumab were the four main chemotherapeutic agents used in the study population. Although only doxorubicin is an anthracycline, all these drugs have been shown to be potentially cardiotoxic [5–7].

### Body composition acquisition and analysis

**Fig. 1:**
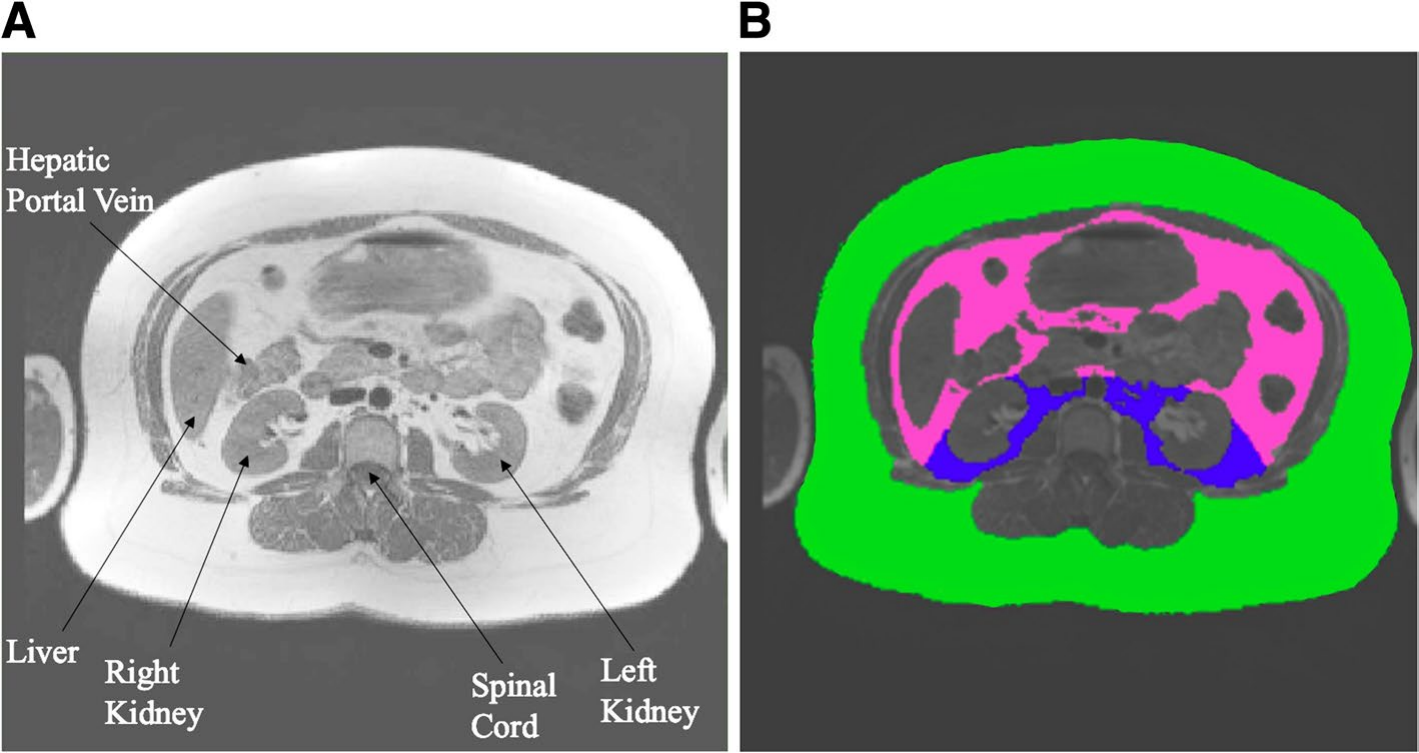
**A** Abdominal MRIs at the second lumbar vertebra were obtained. The hepatic portal vein, liver, kidneys, and spinal cord were visualized in the abdominal MRI. **B** From the image in Fig. 1A, color-coding was used to denote adipose tissue depots. The green represents subcutaneous adipose tissue, while the pink and blue represent intraperitoneal and retroperitoneal adipose tissue, respectively. Intra-abdominal adipose tissue was the combination of intraperitoneal (pink) and retroperitoneal (blue)

Prior to the receipt of cancer treatment, abdominal MRIs were obtained from the study site community hospitals using 1.5 and 3.0 Tesla scanners from various manufacturers according to previously published techniques [8]. Imaging parameters, including a 256 × 256 matrix and 5-mm-thick slice, were identical across all scanners. Briefly, axial slices that encompassed the entire abdomen and included the hepatic portal vein, liver, kidneys, and spinal cord (Fig. 1a) were obtained at the level of the second lumbar vertebra (Fig. 1a). IAT and SAT volumes were drawn and quantified (Fig. 1b) using the TomoVision SliceOmatic software (Magog, QCJ1X 0R4, Canada).

### LVEF Acquisition and analysis

Cardiac MRI exams were obtained to determine the LVEF at baseline and 3-months after treatment initiation. LVEF was determined with a non-contrast 10- to 15-min rapid CMR examination following previously published methods, in which a cine bright blood steady-state free precision technique was used with 128 × 102 matrix, 40 × 32-cm field of view, minimized echo time, maximized flip angle (45 to 70 degrees), 8-mm-thick slice with 2-mm inter-slice gap, and 22-ms temporal resolution [9–11]. A reader blinded to all patient identifiers analyzed cardiac MRI cine slices with QMASS (Medis, Ljubljana-Črnuče, Slovenia). Following identification of the end-diastolic and end-systolic phase images, the endocardium and epicardium were outlined for both images.

### Statistical analysis

Descriptive statistics included the calculation of means ± standard deviations for continuous measures and counts and percentages for categorical measures. Pearson correlations were used to examine associations between 3-month changes in LVEF and baseline BMI, IAT and SAT. A composite variable that considered five CVD risk factors [age over 60 (yes/no), diabetes mellitus (yes/no), smoker (yes/no), coronary artery disease (yes/no), and hypertension (yes/no)] was summed to represent the total number of the CVD risk factors. Pearson correlations were re-estimated, adjusting for the number of CVD risk factors present. Next, we fit a general linear model to examine if IAT (or SAT), coded as above or below the median, was associated with 3-month LVEF change, adjusting for the number of CVD risk factors. Additionally, we added BMI to this general linear model. Correlation between variables was assessed with a correlation matrix. When we examined the potential for multicollinearity among the three variables SAT, IAT, and BMI we noticed that BMI and SAT had a variance inflation factors above 3.0 (3.6 and 3.4, respectively), suggesting collinearity. To further analyze potential impacts of this collinearity, we fit a stepwise regression model which showed that only BMI entered into the regression model, suggesting that it had the highest association with the outcome (3-month LVEF decline) and once that variable was included neither SAT or IAT added any statistically significant impact to the model.

We then examined the impact of IAT (or SAT) on LVEF change stratified by IAT (or SAT) median value while adjusting for CVD risk factors and BMI in a general linear model. We fit an overall general linear model to examine LVEF change by including BMI, IAT, SAT, and number of CVD risk factors as independent variables in the model. For all analyses a *p*-value < 0.05 was considered significant, and analyses were conducted using Statistical Analysis Software.

## Results

### Patient demographics

In total, 213 women were enrolled in the present study. Three patients were lost to follow-up, leaving 210 participants. The women were primarily of non-Hispanic White race with the majority being middle-aged breast cancer patients, about one-third having hypertension, and were receiving potentially cardiotoxic chemotherapy (Table 1). The average 3-month LVEF decline was 2.8 ± 6.1% (Table 2). Average baseline BMI prior to initiating cancer treatment was 29.7 ± 6.0 kg/m^2^, which is displayed alongside average abdominal adipose tissue volumes representing a single slice of abdominal MRI (Table 3).

**Table 1:** Descriptive characteristics [values expressed as *n* (%) or mean ± standard deviation]

	All Patients (*n* = 210)
Age (years)	55.8 ± 11
Heart Rate (bpm)	74 ± 12
Systolic BP (mmHg)	126 ± 17
Diastolic BP (mmHg)	76 ± 10
Race	
White	165 (79)
Black	36 (17)
Asian	2 (1)
Southeast Pacific Islanders	1 (0.5)
Unreported/Unknown	6 (2.5)
Cancer	
Breast	195 (93)
Lymphoma	14 (6.5)
Sarcoma	1 (0.5)
Treatment	
Doxorubicin	87 (41)
Cyclophosphamide	112 (53)
Paclitaxel	94 (45)
Trastuzumab	49 (23)
Average cumulative anthracycline dose (mg/m^2^)	261 ± 115
Breast	234 ± 62
Lymphoma	466 ± 184
Sarcoma	149.6
Cardiovascular disease Risk Factors	
Coronary Artery Disease	6 (3)
Diabetes	19 (9)
Tobacco	57 (27)
Hypertension	71 (34)

*Abbreviations: bpm *beats per minute,* mmHg *millimeters of mercury,* mg/m*^*2*^milligrams per meter squared

**Table 2:** Descriptive statistics for cardiac measures (values are expressed as mean ± standard deviation)

Baseline Left Ventricular End-Diastolic Volume (mL)	119.1 ± 24
3-month Left Ventricular End-Diastolic Volume (mL)	117 ± 24
Baseline Left Ventricular End-Systolic Volume (mL)	45 ± 12
3-month Left Ventricular End-Systolic Volume (mL)	47 ± 13
Left Ventricular Ejection Fraction (%)	
Baseline	62.2 ± 5.5
3 Months	59.5 ± 6.2

**Table 3:** Descriptive statistics for body composition measures at baseline (values are expressed as mean ± SD)

Body Mass Index (kg/m^2^)	29.7 ± 6
Subcutaneous Adipose Tissue (cm^3^)	263.4 ± 122
Intra-Abdominal Adipose Tissue (cm^3^)	127.2 ± 67
Intraperitoneal Adipose Tissue (cm^3^)	94.8 ± 54
Retroperitoneal Adipose Tissue (cm^3^)	31.4 ± 16

### Correlational and regression analysis

**Fig. 2:**
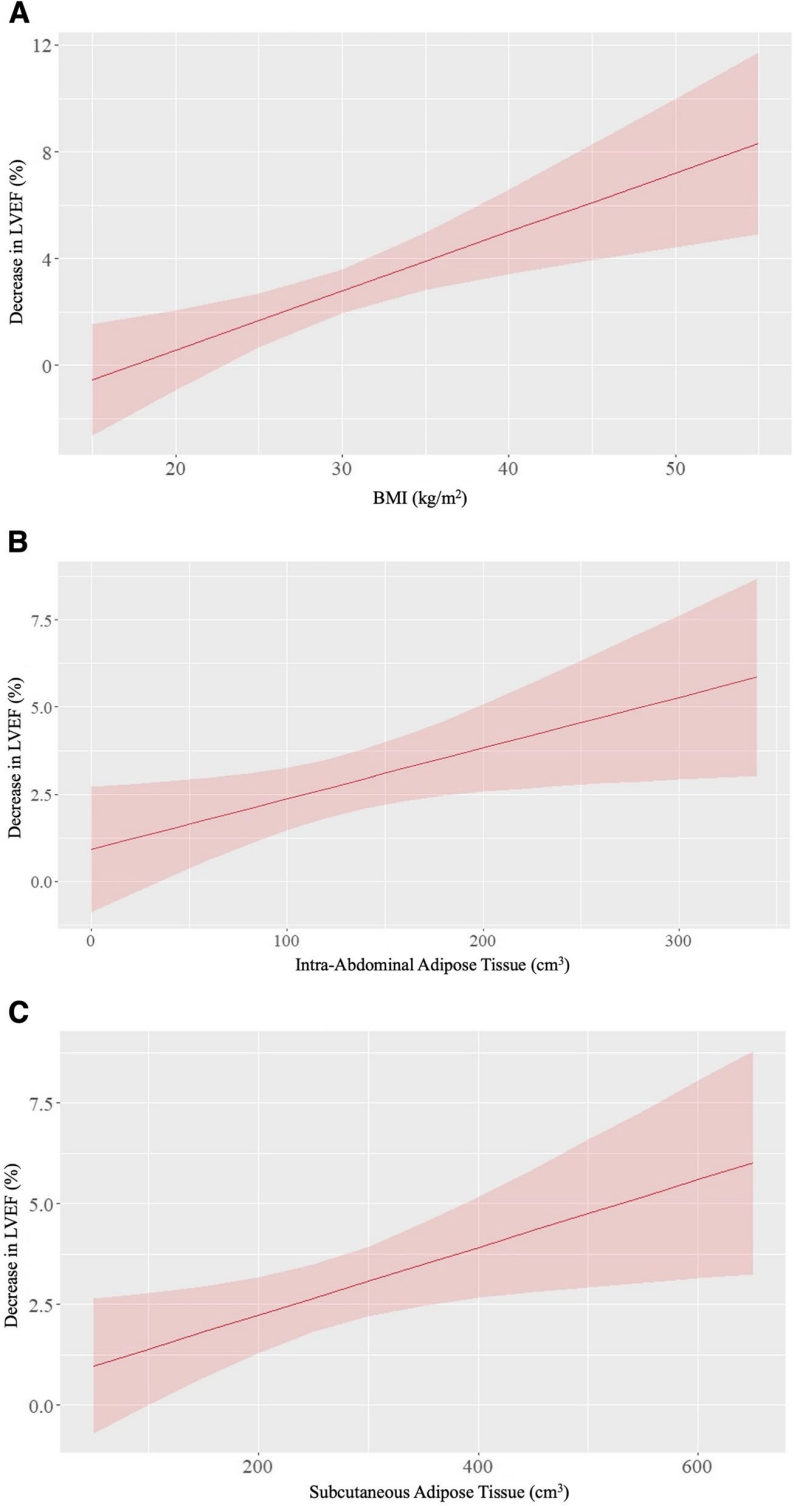
**A** Line graph with body mass index (BMI) in kg/m^2^ on the x-axis and percentage decrease in left ventricular ejection fraction (LVEF) on the y-axis adjusted for cardiovascular disease risk factors (age, tobacco use, diabetes mellitus, hypertension, and coronary artery disease), intra-abdominal adipose tissue, and subcutaneous adipose tissue. As BMI increases, there is a greater 3-month LVEF decrease (β = 0.25, *p* = 0.047). **B** Line graph with Intra-abdominal adipose tissue (cm^3^) on the x-axis and percentage. Decrease in left ventricular ejection fraction (LVEF) on the y-axis adjusted for cardiovascular disease risk factors (age, tobacco use, diabetes mellitus, hypertension, and coronary artery disease), body mass index, and subcutaneous adipose tissue. As intra-abdominal adipose tissue volume increases, there is a greater decrease in 3-month LVEF change (β = 0.005, *p* = 0.48). **C** Line graph with Subcutaneous adipose tissue (cm^3^) on the x-axis and percentage. Decrease in left ventricular ejection fraction (LVEF) on the y-axis adjusted for number of cardiovascular disease risk factors (age, tobacco use, diabetes mellitus, hypertension, and coronary artery disease), body mass index, and intra-abdominal adipose tissue. As subcutaneous adipose tissue volume increases, there is a greater decrease in 3-month LVEF change (β = -0.004, p = 0.56)

Table 4 displays correlation analyses between body composition and change in LVEF. In an unadjusted analysis, the 3-month change in LVEF positively correlated with baseline BMI (β = 0.23, *p* = 0.001), IAT (β = 0.15, *p* = 0.025), and SAT (β = 0.17, *p* = 0.016). To account for the potential impact of CVD risk factors such as age, tobacco use, diabetes mellitus, hypertension, and coronary artery disease) on the relationship between body composition and LVEF change, we created a multivariable model adjusting for CVD risk factors. In this adjusted model that included the number of CVD risk factors, BMI, IAT, and SAT, only BMI remained associated with 3-month LVEF change (*p* = 0.047; Fig. 2). Respective β-coefficients and *p*-values are summarized in Table 4. We note that BMI and SAT are highly correlated with one another in the model adjusting for CVD (*r* = 0.83), and therefore both variables may have individually been predictive of the outcome, but our data suggest that BMI enters the model preferably relative to SAT.

**Table 4:** Correlations between body composition measures and 3-month decrease in LVEF

	β (unadjusted)	*p*-value	β (adjusted)	*p*-value
Body Mass Index	0.22	0.001	0.25	0.047
Intra-Abdominal Adipose Tissue	0.014	0.025	0.005	0.48
Subcutaneous Adipose Tissue	0.008	0.016	-0.004	0.56

The ‘adjusted’ column displays results from a multivariable model adjusting for cardiovascular disease risk factors (age, tobacco, diabetes mellitus, hypertension, coronary artery disease), body mass index, intra-abdominal adipose tissue, and subcutaneous adipose tissue

In the case that LVEF is a categorical variable with LVEF decline > 10% as the threshold, we found that BMI continued to be the only significant factor affecting 3-month LVEF change (*p* = 0.44). Moreover, 3-month change in BMI is also a predictor of 3-month LVEF change (*p* = 0.004, *r* = 4.7%), however baseline BMI is a slightly stronger predictor of 3-month LVEF change (*p* = 0.001, *r* = 5.1%). Additionally, in our study sample the relationship between hypertension (*p* = 0.15), as well as diastolic (*r*^2^ = 0.0024, *p* = 0.59) and systolic (*r*^2^ = 0.0093, *p* = 0.29) blood pressure, race (*p* = 0.7), type of malignancy (*p* = 0.17), anthracycline status (*p* = 0.2), and CVD risk factors (*p* = 0.8) with 3-month LVEF change was insignificant.

## Discussion

The main findings of this cohort study of women scheduled to receive potentially cardiotoxic chemotherapy for breast, lymphoma, or soft tissue sarcoma cancer include: a) BMI, IAT, and SAT are individually associated with 3-month LVEF declines, and b) after accounting for CVD risk factors and combining BMI with IAT and SAT, only BMI remains independently associated with 3-month LVEF decline. In this later analysis, the fitted model explained about 5.5% of the variability, suggesting that substantial variability is left to be explained by other characteristics among individuals with BMI elevations that may include physical inactivity, psychosocial stress, diet, sleep disorders, or vitamin D deficiency. Combined, these results suggest that factors other than the presence of IAT or multiple traditional CVD risk factors are related to LVEF declines in women with elevated BMI receiving potentially cardiotoxic chemotherapy for breast, lymphoma, or sarcoma cancer.

In this prospective cohort study, we enrolled 210 women, with an average age of 55.8 ± 11 years, who had been diagnosed with breast (93%), lymphoma (14%), or sarcoma (1%) cancer. As shown in Table 1, most patients were of White race, with 34% having hypertension, 3% having coronary artery disease, 9% having diabetes, and 27% reporting current tobacco use- aligning closely with the demographic profile seen in comparable studies [4, 11]. The average 3-month change in LVEF was -2.8 ± 6.1%, a figure consistent with similar studies [11]. However, the average baseline BMI in our study was slightly higher at 29.7 ± 6 kg/m^2^ compared to 25.6 kg/m^2^ and 27 kg/m^2^reported in similar investigations [12, 13]. Thus, across many demographics, the women reported in this study were similar to those in others, with the exception that the BMI was higher than previous reports.

Our study sample was mainly composed of breast cancer patients (93%), and the average cumulative anthracycline dose for the breast cancer patients in our study was roughly half of the average cumulative anthracycline dose for lymphoma cancer patients in our study. Additionally, breast cancer is typically treated with lower cumulative anthracycline dose than lymphoma cancer [14]. Prior studies included a smaller ratio of breast cancer patients, which indicates that chemotherapy dose may play a role in the relationship between adipose tissue depots and cardiotoxicity [4].

As demonstrated in Fig. 2A, after accounting for CVD risk factors, IAT, and SAT, BMI correlated with the 3-month LVEF decline (*p* = 0.047). Previously, we noted that intraperitoneal fat, but not BMI, was associated with 24-month LVEF declines in men and women receiving chemotherapy (*r* = -0.33, *p*= 0.02) [4]. The current study, however, included only women. It’s important to note that body fat percentage is higher in women than in men, and thus the interaction between hormones, such as estrogen, and BMI influencing 3-month LVEF change should be further investigated in a study with direct comparison to males of similar BMI, age, and anthracycline dose [15, 16].

In our previous study, we determined a relationship between IAT and 3-month LVEF decline, and in univariate analysis for this study, this relationship persisted. Interestingly, however, in these large BMI individuals, we found that SAT was also associated with 3-month LVEF decline in univariate analysis.

After accounting for traditional CVD RFs in a multivariable analysis, we determined that only IAT and SAT did not correlate with 3-month LVEF decline, implying that in women with a large BMI, factors other than IAT or SAT are responsible for 3-month LVEF decline. Other possible associated factors in women with elevated BMI could be lifestyle and behavioral CVD risk factors, such as physical inactivity [17–22], psychosocial stress [23, 24], diet [25, 26], sleep disorders [27–29], and vitamin D deficiency [30–32].

Moreover, we previously noted that SAT was associated with preservation of LVEF during cancer treatment, and interestingly had an inverse relationship with 24-month LVEF decline. However, in our current study, we found no significant relationship between SAT and 3-month LVEF decline in our multivariable analysis.

Our study had limitations. First, the majority of our study participants were diagnosed with breast cancer. Thus, the findings may not be generalizable to all cancer types. Second, the average baseline BMI in our study was slightly higher than similarly designed studies, and therefore the findings may only be applicable to patients with higher BMIs. Finally, our study does not account for lifestyle and behavioral risk factors, thus warranting further studies that explore the effects of these modifiable cardiovascular risk factors on the relationship between BMI and 3-month LVEF decline.

## Conclusions

In this multicenter study, we determined that in women with breast, lymphoma, or sarcoma cancer, traditional CVD risk factors (age, tobacco, diabetes mellitus, hypertension, coronary artery disease), IAT, and SAT do not fully explain the relationship between BMI and 3-month LVEF decline. Upon adjusting for CVD risk factors, IAT, and SAT, BMI remained associated with 3-month LVEF decline. This study population was unique in that it was all women, the majority of which were breast cancer patients, with lower dose chemotherapy and higher-than-average BMIs. Therefore, further studies should be conducted to determine the impacts of lifestyle and behavioral CVD risk factors, such as physical inactivity, psychosocial stress, diet, sleep disorders, and vitamin D deficiency, on the relationship between BMI and 3-month LVEF decline.

## Data Availability

The data analyzed during the current study are available from the corresponding author upon reasonable request.

## Abbreviations

BMI: Body mass index
CVD: Cardiovascular disease
IAT: Intra-abdominal adipose tissue
IP: Intraperitoneal
LVEF: Left ventricular ejection fraction
MRIs: Magnetic resonance images
RP: Retroperitoneal
SAT: Subcutaneous adipose tissue
